# Zoledronic acid impairs stromal reactivity by inhibiting M2-macrophages polarization and prostate cancer-associated fibroblasts

**DOI:** 10.18632/oncotarget.9497

**Published:** 2016-05-20

**Authors:** Giuseppina Comito, Coral Pons Segura, Maria Letizia Taddei, Michele Lanciotti, Sergio Serni, Andrea Morandi, Paola Chiarugi, Elisa Giannoni

**Affiliations:** ^1^ Department of Experimental and Clinical Biomedical Sciences, University of Florence, 50134 Florence, Italy; ^2^ Tuscany Tumor Institute and “Center for Research, Transfer and High Education DenoTHE”, 50134 Florence, Italy; ^3^ Department of Urology, University of Florence, Careggi Hospital, Urologic Clinic San Luca, 50100 Florence, Italy

**Keywords:** zoledronic acid, prostate cancer, cancer-associated fibroblasts, macrophages, mevalonate pathway

## Abstract

Zoledronic acid (ZA) is a biphosphonate used for osteoporosis treatment and also proved to be effective to reduce the pain induced by bone metastases when used as adjuvant therapy in solid cancers. However, it has been recently proposed that ZA could have direct anti-tumour effects, although the molecular mechanism is unknown. We herein unravel a novel anti-tumour activity of ZA in prostate cancer (PCa), by targeting the pro-tumorigenic properties of both stromal and immune cells. Particularly, we demonstrate that ZA impairs PCa-induced M2-macrophages polarization, reducing their pro-invasive effect on tumour cells and their pro-angiogenic features. Crucially, ZA administration reverts cancer associated fibroblasts (CAFs) activation by targeting the mevalonate pathway and RhoA geranyl-geranylation, thereby impairing smooth muscle actin-α fibers organization, a prerequisite of fibroblast activation. Moreover, ZA prevents the M2 macrophages-mediated activation of normal fibroblast, highlighting the broad efficacy of this drug on tumour microenvironment. These results are confirmed in a metastatic xenograft PCa mouse model in which ZA-induced stromal normalization impairs cancer-stromal cells crosstalk, resulting in a significant reduction of primary tumour growth and metastases. Overall these findings reinforce the efficacy of ZA as a potential therapeutic approach to reduce cancer aggressiveness, by abrogating the supportive role of tumour microenvironment.

## INTRODUCTION

Inflammation is now acknowledged as a hallmark of cancer and several tumour-associated cells are active players in promoting a pro-inflammatory microenvironment, including cancer associated fibroblasts (CAFs) and tumour-associated macrophages (TAMs). These accessory cells establish a malignant cross-talk with cancer cells that affects the behaviour of each other, ultimately allowing cancer to acquire aggressive features [1].

CAFs, one of the major components of tumour microenvironment, have been reported to induce epithelial-to-mesenchymal transition (EMT), invasiveness, resistance to *anoikis* and chemotherapeutic agents, as well as stem-like features of cancer cells, thereby contributing to metastatic spread [2–4]. CAFs also cooperate with tumour cells in fostering *de novo* angiogenesis [5]. In addition, CAFs establish a metabolic cross-talk with cancer cells, an acknowledgment prerequisite of highly aggressive tumours. Particularly, prostate cancer (PCa) cells meet their energetic and anabolic requirements by exploiting the lactate produced by glycolysis-dependent CAFs [6, 7].

CAFs have also been reported to induce the establishment of a pro-inflammatory microenvironment, by concurring to monocyte recruitment to tumour site and polarization into the pro-tumoral subset of M2 macrophages in a stromal derived growth factor-1-dependent manner [8]. In turn, M2 macrophages support malignant progression by promoting angiogenesis and the establishment of an immunosoppressive microenvironment [9, 10]. In keeping, switch of M1 macrophages, endowed with immune stimulating and anti-tumour activities, towards the M2 phenotype is associated with a worse clinical prognosis and high grade of malignancy [11, 12]. Recent studies also demonstrate that M2 macrophages are able to modulate stromal reactivity, by inducing fibroblast activation [8]. This interplay between CAFs and M2 macrophages promotes (i) tumour cell motility, hence favouring cancer cells to leave the primary tumour site and (ii) the activation of both endothelial cells and their bone-marrow-derived precursors to drive *de novo* angiogenesis, a step that facilitate metastatic dissemination [8].

Zoledronic acid (ZA) is a FDA-approved third-generation amino-bisphosphonate that has been used as adjuvant therapy to reduce skeletal related events (SREs) and pain associated with bone metastases in several types of cancer, including PCa [13]. Additionally, ZA has been found to have a direct anti-tumour efficacy. Indeed, ZA inhibits cell proliferation and induces apoptosis in human leukemic cell lines [14]. In addition, ZA directly target endothelial progenitor cells interfering with their differentiation, thus impairing their supportive role in cancer cells escape from primary sites [15]. More recently, ZA has been shown to improve immunesurveillance against tumour and to modulate macrophage differentiation, opening new possibilities for its therapeutic application [16]. However, the molecular mechanisms behind the anti-cancer response of ZA and its effects on non-cancerous stromal cells, remains yet largely unclear and need to be clarified.

Here, we report that in PCa cellular models ZA impairs both M2 macrophages differentiation and CAF activation, thus impairing their supportive roles in PCa tumour progression. This suggests that the benefit of ZA in the therapy of PCa-bearing patients, potentially goes beyond the simple skeletal/bone symptoms treatment, but is enlarged to regulation of stromal inflammatory events, with a consequent impact on cancer cells malignancy.

## RESULTS

### ZA treatment impairs M2 macrophages polarization

We have recently reported that PCa cells recruit monocytes and polarize them towards an M2 macrophages phenotype, which in turn promote cancer cell invasiveness [8]. Since ZA has been proposed to have anti-tumoral and immunomodulatory properties [17], we aim at investigating the effect of the compound on this feed-forward loop between cancer and immune cells. Monocytes isolated from normal human blood donors buffy coat were treated with macrophage colony-stimulating factor (MCSF) to promote differentiation into macrophage, enabling them to proliferate and later acquire a specific polarization into M1, M2 or M2-like macrophages upon stimulation with LPS+IFN-γ, IL-4 or CM from PC3, respectively. Of note, treatment to polarize towards a M1, M2 or M2-like phenotype by means of specific treatment During the differentiation period, ZA was administrated at different doses (Supplementary Figure S1A).

**Figure 1:**
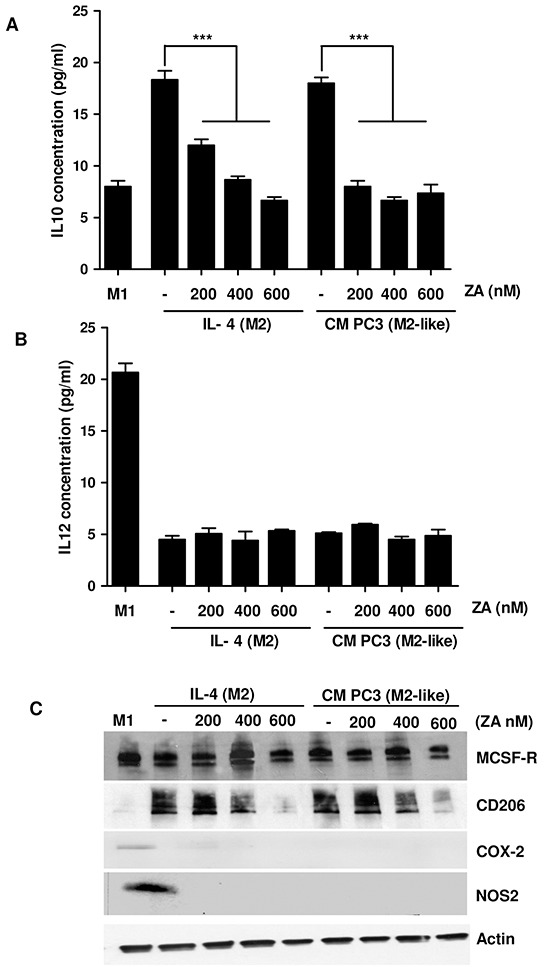
ZA suppresses monocyte differentiation toward M2 macrophages **A, B.** Human monocytes isolated from normal donor buffy coat were cultured for 7 days with M-CSF (50ng/ml). Differentiation toward the M1 phenotype was induced by treatment with LPS (100 ng/ml) and IFNγ (100 ng/ml) for 24h. M2 macrophages were polarized by stimulating with IL-4 (20 ng/ml) for 24h. Alternatively, M2-like macrophages were obtained by treating isolated monocytes with CM from PC3 for 7 days. During differentiation macrophages were treated with different concentrations of ZA and the levels of IL-12 or IL-10 were measured by ELISA test. 1-way ANOVA, Dunnett's corrected, ***p< 0,001 *vs* untreated. **C.** Cells treated as in A were lysed and the expression of M-CSFR, COX-2, CD206, NOS2 and actin was evaluated by immunoblots.

To analyse if ZA treatment can impact on macrophages polarization, we evaluated the production of IL-10 and IL-12. Indeed, while M1 macrophages are characterized by IL-12^high^/IL-10^low^ phenotype, M2 macrophages show an IL-12^low^/IL-10^high^ production [18]. The results demonstrate that treatment with ZA reduces IL-10 expression without affecting IL-12 levels, suggesting a drug-dependent impairment of M2 polarization, without a conversion towards the M1 phenotype (Figure 1A-1B). Notably, ZA appears to be effective at the lower dose of 200 nM, without any significant dose-dependent effect. To further analyse the M1/M2 subtype modulation upon ZA administration, we analysed the expression of MCSF receptor (MCSF-R), in order to confirm the acquisition of the differentiated phenotype, CD206 as a cell surface marker for M2 polarized macrophages and cycloxigenase-2 (COX-2) and iNOS as markers for the M1 subtype. MCSF-R is highly expressed by macrophages cultured with MCSF, as well as by macrophages incubated with CM from PC3 or treated with ZA. In contrast, only M1 macrophages express high level of COX-2 (Figure 1C). These data highlight that ZA can reduce the pool of pro-tumoral M2-polarized macrophages, without promoting a real unbalance towards the anti-tumoral M1-polarized macrophages.

### ZA-treated M2-polarized macrophages negatively affect invasiveness of PCa cells

**Figure 2:**
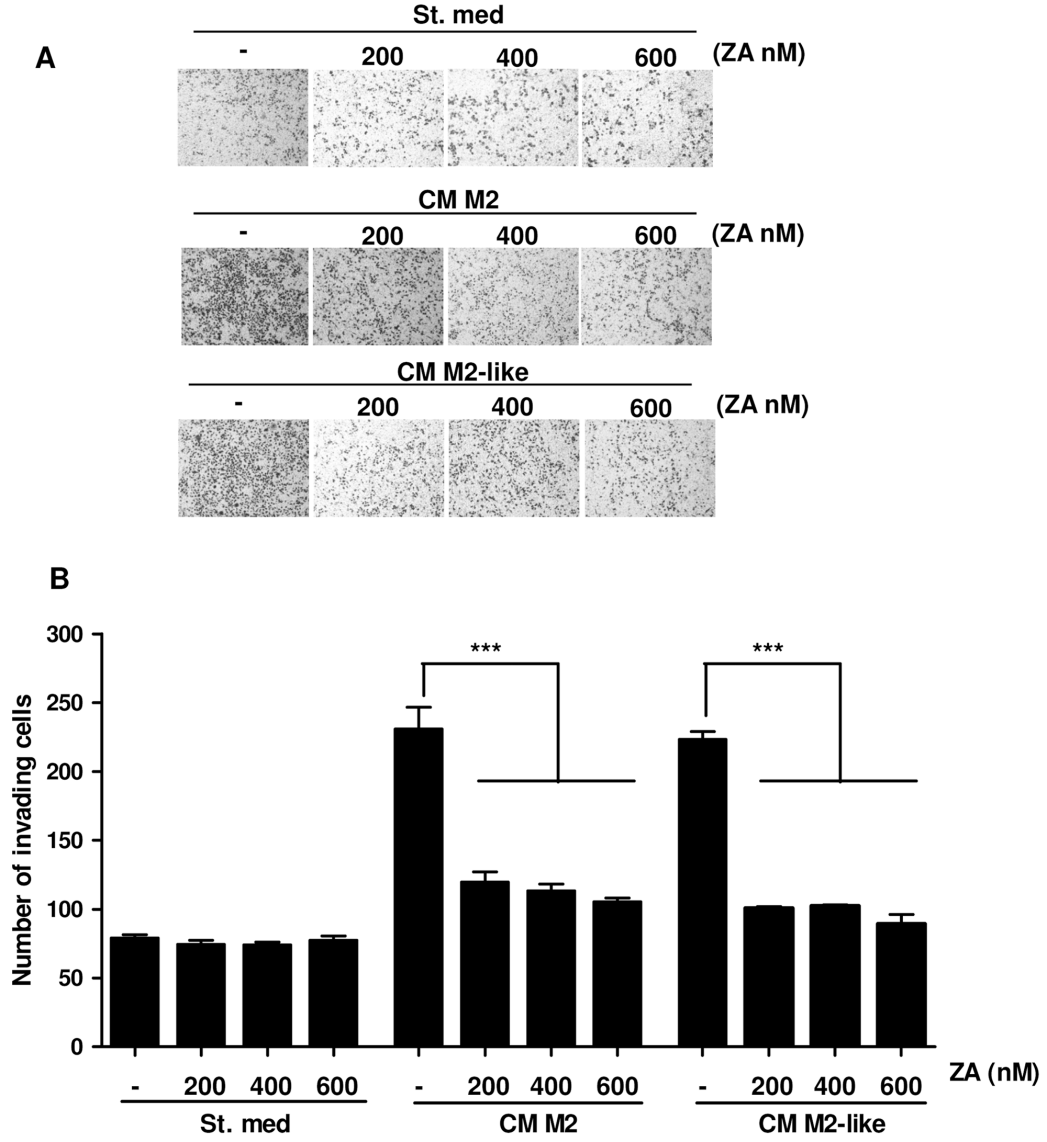
M2 and M2-like macrophages-dependent increase of PC3 cells invasiveness is impaired by ZA treatment **A.** Monocytes were differentiated for 7 days with M-CSF and then polarized into M2 macrophages by stimulating with IL-4 for 24h. Alternatively, monocytes were stimulated with CM from PC3 for 7 days to obtain M2-like macrophages. ZA was administrated during differentiation at different concentrations and then macrophages were serum-starved for 48h to obtain the corresponding CM. PC3 cells were incubated for 24h with CM from the above differentiated macrophages (treated or not with ZA), or serum starved as a control, and then allowed to invade toward medium containing 10% serum as chemoattractant for additional 24 h. (St. Med. Starvation Medium) **B.** Invading cells were counted and the mean of six randomly chosen fields was plotted in the bar graph. 1-way ANOVA, Dunnett's corrected, ***p< 0,001 *vs* untreated.

We have reported that M2 and M2-like macrophages are able to increase cancer cells malignancy [8]. To evaluate the effects of both M2 or M2-like macrophages treated with ZA on PC3 cells motility, CM from the above-treated macrophages were collected and administered to PCa cells for 24h. An invasion assay was then performed. In agreement with our previous data, M2 and M2-like macrophages elicit a pro-invasive effect on PC3 cells, which is significantly impaired by ZA administration (Figure 2A-2B). Importantly, PC3 cell survival was unaffected by ZA treatment, therefore excluding an effect on cell viability rather than on cell motility (Supplementary Figure S2). These data reveals that ZA-dependent inhibition of M2 polarization leads to an indirect impairment of tumour cell invasion.

### ZA hinders the angiogenic response induced by M2 and M2-like macrophages

**Figure 3:**
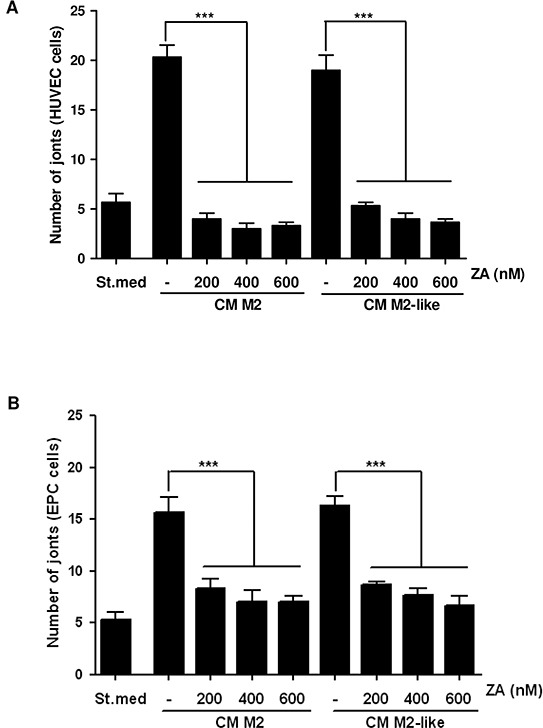
Treatment with ZA inhibits the M2/M2-like macrophages-induced pro-angiogenic effect M2 and M2-like macrophages were obtained by treating monocytes for 7 days with M-CSF (then treated with IL-4 for additional 24h) or with CM from PC3, respectively. M2 and M2-like macrophages were treated with different concentrations of ZA during differentiation and then were serum-starved for 48h to obtain the corresponding CM. HUVEC **A.** and EPC **B.** cells were treated with CM from the above differentiated macrophages (treated or not with ZA) and *in vitro* angiogenesis was evaluated by capillary morphogenesis assay. The number of joints was quantified and plotted in the bar graph. 1-way ANOVA, Dunnett's corrected, ***p< 0,001 *vs* untreated.

We demonstrated that M2 macrophages contribute to increase tube-like structures formation in EPCs and HUVECs, suggesting a key role of immune cells in driving tumour vascularization [8]. We therefore investigated whether the contact with ZA-treated M2/M2-like-polarized macrophages, could affect the angiogenic features of endothelial cells. We treated HUVECs or EPCs with CM from the differently treated macrophages and assayed their ability to form tube-like structures *in vitro*. We showed that upon ZA-treatment there is a significant reduction of capillary-like structures assembly, suggesting that the drug, through the impairment of M2 polarization, may indirectly affect *de novo* intratumoral angiogenesis (Figure 3 and Supplementary Figure S3).

### ZA reverts CAF-activated phenotype and their pro-invasive features

**Figure 4:**
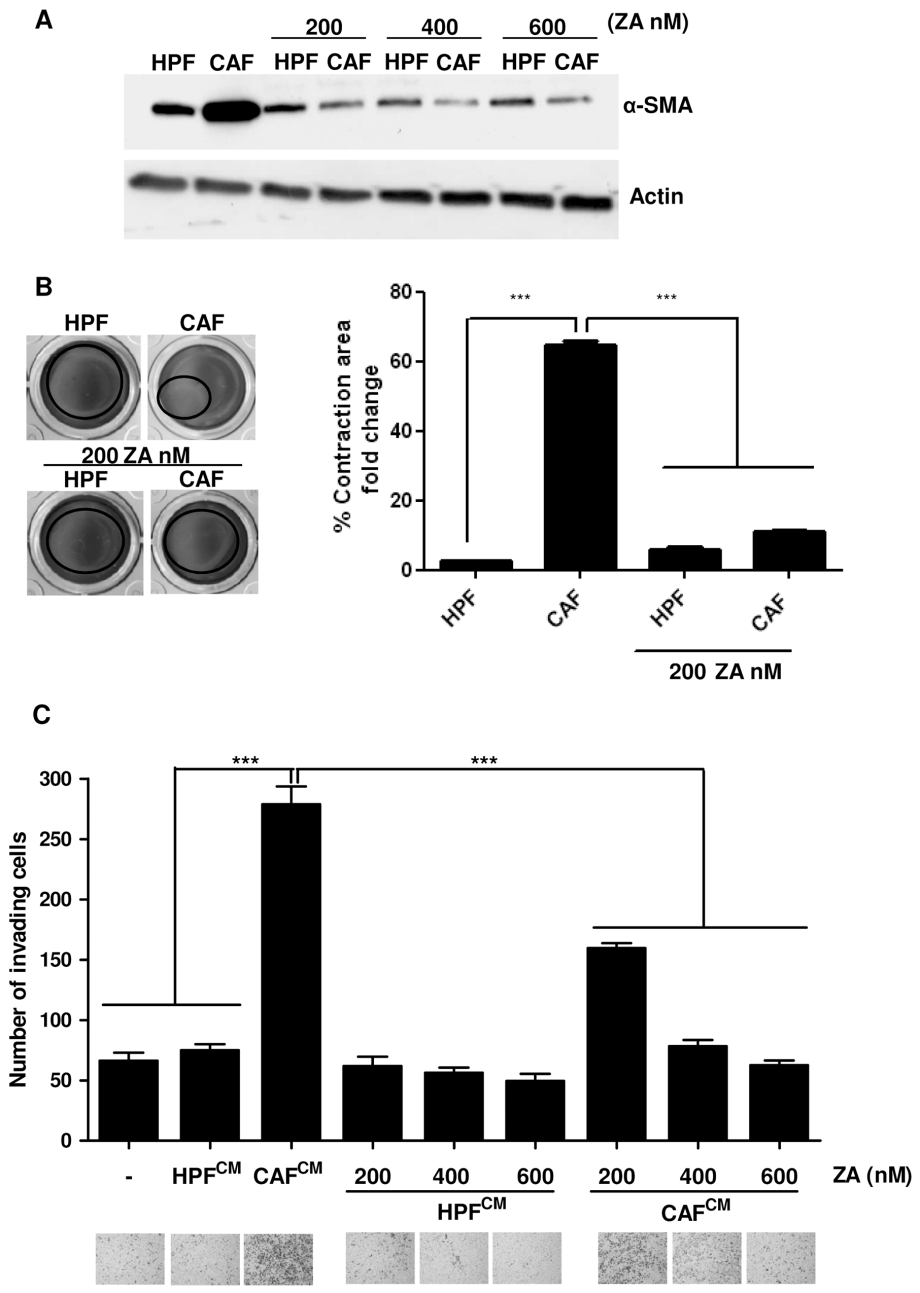
ZA administration reverts CAF activation and impairs their pro-invasive effects on cancer cells **A.** Subconfluent HPFs and CAFs were serum starved and treated for 5 days with different concentration of ZA. α-SMA was analyzed by immunoblot as a marker for fibroblast activation. Actin was used as loading control. **B**. HPFs and CAFs were treated in serum-free medium with ZA 200nM for 5 days and after were placed in DMEM supplemented with collagen. Serum-starved HPFs and CAFs were used as control cells. Contraction of the collagen discs is expressed as relative collagen area from ST group. 1-way ANOVA, Bonferroni's corrected***p< 0,001 *vs* CAF. **C.** PC3 cells were incubated for 24 h with CM from HPFs and CAFs treated as in A and then allowed to invade for additional 24 h toward medium containing 10% serum as chemoattractant. Invading cells were counted and the mean of six randomly chosen fields was plotted in the bar graph. 1-way ANOVA, Dunnett's corrected, ***p< 0,001 *vs* CM CAF.

To investigate the potential effects of ZA on other cellular components of tumour microenvironment, we focused our interest on stromal fibroblasts, since the achievement of aggressive features of cancer cells is strongly dependent on their contact with surrounding CAFs [2, 3]. Normal fibroblasts (HPFs) and CAFs were treated with increasing concentrations of ZA for 5 days. The evaluation of alpha smooth muscle actin (α-SMA) expression, a reported marker of fibroblasts activation (Figure 4A) and the quantification of collagen contractility, an established feature of activated fibroblasts (Figure 4B), reveal that ZA administration completely reverts the activated phenotype of CAFs. To confirm the ZA-dependent conversion of CAFs towards a non-activated state, we explored whether ZA-treated CAFs were still able to promote cancer cell motility. PC3 cells were conditioned with CM obtained from HPF and CAF treated as above, and tested for their migratory abilities (Supplementary Figure S1B). Crucially, ZA-treatment abolishes CAF ability to enhance PC3 cell invasiveness (Figure 4B-4C). Comparable results were obtained with DU145, an additional PCa cell line (Supplementary Figure S4).

These data reveals that ZA impairs CAF activation and therefore their supportive role in PCa malignancy.

### ZA hinders fibroblast activation through the inhibition of RhoA-GTPase activity

**Figure 5:**
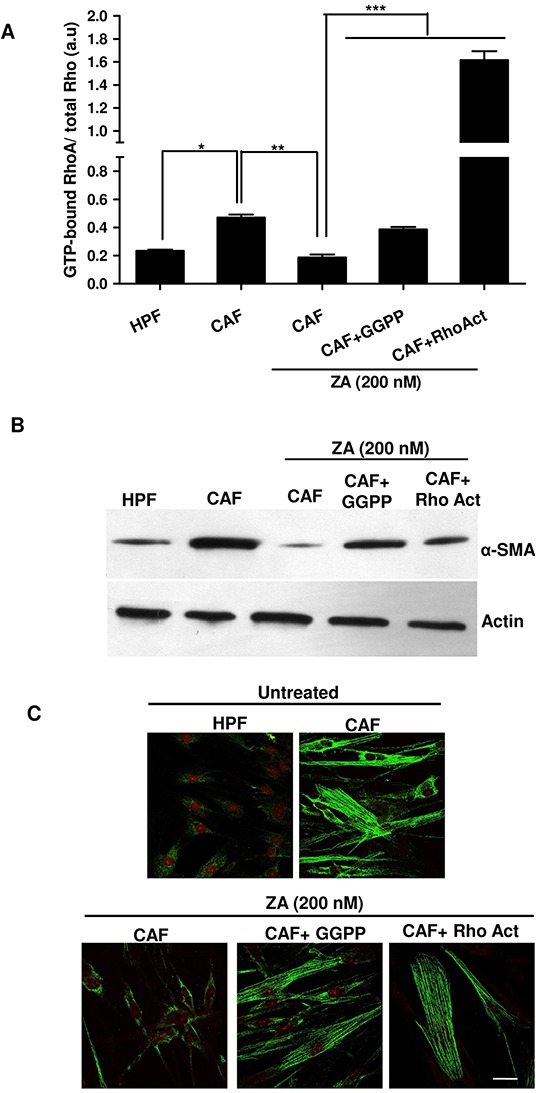
ZA inhibits RhoA prenylation/activation, thereby abrogating α-SMA organization and CAF activation **A.** CAFs were treated in serum-free medium with ZA 200nM for 5 days alone or in combination with geranylgeranyl pyrophosphate 20 μM (ZA+GGPP) or Rho Activator (ZA+RhoAct). Serum-starved HPFs and CAFs were used as control cells. ActiveGTP-bound RhoA was quantified by Rhotekin pull-down assay and normalized by total RhoA immunoblot upon densitometric analysis. 1-way ANOVA, Bonferroni's corrected, *p< 0,05 *vs* HPF, **p< 0,01 *vs* CAF, ***p< 0,001 *vs* CAF+ ZA. **B-C.** Fibroblasts were treated as in A. α-SMA was quantified by both immunoblot (B) and confocal microscopy. Bar, 50 μm (C).

It has been reported that ZA inhibits the active site of the enzyme farnesyl pyrophosphate synthase in the mevalonate pathway, resulting in reduced levels of isoprenoids, including farnesyl pyrophosphate (FPP) and geranyl-geranyl pyrophosphate (GGPP), required for the prenylation of GTPase signalling proteins (i.e. Rac, Rho, Cdc42). This lipid modification is essential for the small GTPases family to achieve their active state and to ensure cytoskeletal rearrangements and dynamic cell shape remodelling [19]. To gain insight into the possible molecular mechanism responsible for ZA-mediated reversion of CAFs activated state, we focused on the Rho family GTPases and we examined whether ZA was able to affect RhoA activity by reducing its geranyl-geranylation. To evaluate RhoA activity, CAFs were treated in the presence or absence of 200nM ZA, the lower dose of the drug that is effective in the reversion of CAFs activated phenotype, and then a pull-down assay of active GTP-bound RhoA was performed. Our results indicate that ZA treatment reduces RhoA activity, while the exposure to GGPP or to the Rho activator calpeptin, concomitantly to ZA administration, restores the GTP-bound active state (Figure 5A). In addition, analysis of α-SMA expression in our experimental setting underlines that RhoA signalling is critical for fibroblast activation. Indeed, ZA-mediated prevention of RhoA activation strongly reduces α-SMA expression, while the addition of GGPP or Rho activator avoids α-SMA down-regulation, thus overcoming ZA-mediated mevalonate pathway inhibition (Figure 5B).

To corroborate these data, we also analysed α-SMA stress fibers organization by confocal microscopy. While ZA-treated cells showed a faint signal with almost no stress fibers, GGPP or Rho activator administration recovered pronounced α-SMA fibers formation (Figure 5C), suggesting the role of ZA in impairing RhoA-mediated α-SMA organization and stabilization of CAF phenotype.

### ZA abrogates macrophage-dependent fibroblast activation

**Figure 6:**
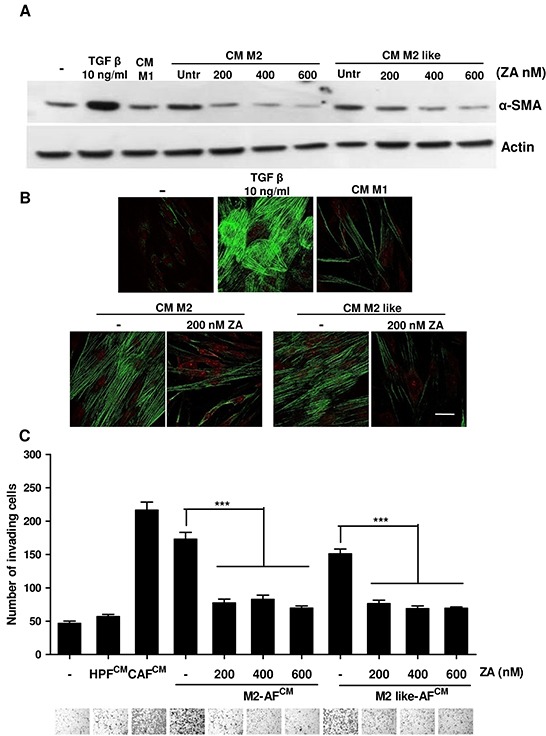
ZA counteracts macrophage-dependent fibroblasts activation **A.** M2 and M2-like macrophages were obtained by treating monocytes for 7 days with M-CSF (then treated with IL-4 for additional 24h) or with CM from PC3, respectively. M2 and M2-like macrophages were treated with different concentrations of ZA during differentiation and then were serum-starved for 48h to obtain the corresponding CM. Subconfluent HPFs were treated for 24 h with CM from the above differentiated macrophages (treated or not with ZA) or with 10 ng/ml TGF-β1 as a positive control, and serum starved for additional 24 h. Fibroblasts were lysed and immunoblots for α-SMA and actin were performed. **B**. M2 and M2-like macrophages were obtained as in A and were treated with ZA 200nM during differentiation and then were serum-starved for 48 h to obtain the corresponding CM. Subconfluent HPFs were treated for 24 h with CM from the above differentiated macrophages (treated or not with ZA) or with 10 ng/ml TGF-β1 as a positive control, α-SMA was quantified by confocal microscopy. Bar, 50 μm. **C.** PC3 cells were incubated for 24 h with CM from HPFs treated as in A (CM M2 AFs or CM M2-like AFs; AFs: Activated fibroblasts) and then allowed to invade for additional 24 h toward medium containing 10% serum as chemoattractant. Invading cells were counted and the mean of six randomly chosen fields was plotted in the bar graph. Representative photographs for each sample are shown under the corresponding bar. 1-way ANOVA, Dunnett's corrected, ***p< 0,001 *vs* untreated.

We recently demonstrated that M2 macrophages are able to activate healthy HPFs into CAFs, endowing them with supportive roles for PCa cells [8]. To better clarify the possible role of ZA in the complex interdependency among the different components of tumour microenvironment, we analysed the effects of ZA-treated M2-polarized macrophages on fibroblast activation. HPFs were incubated with CM from M2 or M2-like-macrophages, with or without different concentration of ZA. Fibroblast activation was then analysed by the evaluation of α-SMA expression (Figure 6A), stress fibers organization (Figure 6B) and by assessing their pro-invasive effect on PCa cells (Figure 6C). As expected, while M2 and M2-like macrophages are active in eliciting fibroblast activation and enhancement of PC3 invasiveness, the exposure to ZA strongly prevents these effects. Therefore, ZA is not only capable to revert the activated state of CAFs, already engaged with pro-aggressive roles, but it is also effective in preventing the M2 macrophages-mediated activation of normal fibroblast, thereby strengthening the broad inhibitory function of this drug on tumour microenvironment.

### ZA prevents CAF-induced enhancement of PCa cell tumorigenicity and lung colonization *in vivo*

**Figure 7:**
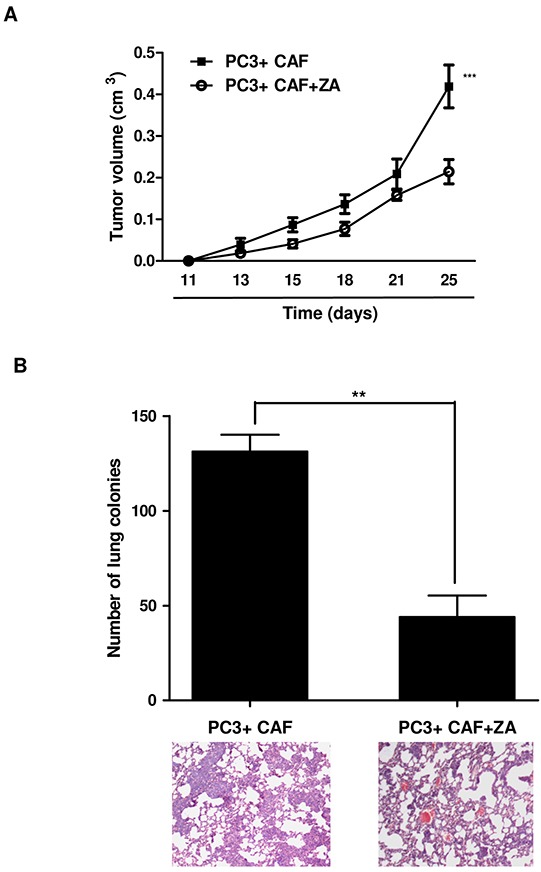
ZA *in vivo* administration prevents tumor growth and lung metastatic dissemination **A.** A mixture of 1 × 10^6^ PC3 cells and 0.5×10^6^ CAFs were subcutaneously injected in both the lateral flanks of SCID bg/bg mice. Mice were treated once a week with intraperitoneal injection of PBS (control mice) or 100 μg/kg of ZA, for 6 weeks (n=5 per group), starting the treatment 24 h after cell injection. The volume of the primary tumour for control or ZA-treated mice was reported at different time points. 2-way ANOVA, Bonferroni's corrected ***p<0,001 *vs* untreated **B.** Mice were sacrificed after 6 weeks. Lungs were inspected and the number of micrometastases were counted and plotted. Representative paraffin-embedded lung tissue sections, stained with hematoxylin/eosin, are shown. Student t-test, **p<0,0036 *vs* PC3+CAF.

As previously reported, CAF conditioning improves tumour growth in xenograft models and promotes lung colonization and metastases formation [2]. According to our *in vitro* results, we aimed to assess whether ZA could be able to neutralize the effects induced by CAFs on tumour growth and metastatic spread *in vivo*. As previously reported, we used PC3 cells in limiting conditions in order to ensure their *in vivo* growth and metastatic spread only when co-injected with CAFs [2, 3]. In particular, a mixture of CAFs with PC3 cells (2:1 ratio) was subcutaneously injected into the lateral flanks of SCID bg/bg mice. Mice received repeated courses of intraperitoneal injection of ZA (100 μg/kg) once a week. We observed that subcutaneous tumours of CAFs/PC3 injected mice showed higher rate of tumour growth compared to ZA-treated tumours (Figure 7A). In addition, histological examination of lungs of tumour-bearing mice revealed the presence of spontaneous micrometastases in all the mice injected with the mixture CAFs/PC3. Conversely, ZA-treated animals exhibited a significant reduction in the number of metastatic areas (Figure 7B).

Comparable results were obtained with DU145 cells (Supplementary Figure S5). These data suggest that the clinical efficacy of ZA could be ascribed to its ability to rebuild a neutral microenvironment which counteracts tumour growth and metastatic spread.

## DISCUSSION

ZA is a bisphosphonate now approved for use in the treatment of cancer-associated bone disease, preventing osteoclast-mediated bone breakdown. Indeed, ZA has been shown to effectively reduce the risk of skeletal-related events in patients with metastatic PCa [20], multiple myeloma [21] and breast cancer [20, 22, 23]. ZA has also recently been tested as an additive therapeutic for early-stage breast cancer [24–26].

In addition to its standard application in the adjuvant treatment of symptomatic bone metastases, several evidence about a direct anti-tumour effect of ZA are emerging [27]. It has been reported that bisphosphonates possess calcium-chelating properties, responsible for the bone-binding properties of these compounds. Since the dysregulation of Ca^2+^ homeostasis has been suggested as an important event in driving the enhancement of malignant traits, such as cell proliferation, migration and invasion [28], as well as tumor vascularization [29], the ZA-dependent regulation of calcium homeostasis may be responsible, at least in part, for its anti-tumor activity. Notably, ZA also attracts interest as a potent immunomodulator, which can be exploited in cancer immunotherapy. Recently, Coscia and colleagues reported that administration of clinically compatible doses of ZA is an effective way of targeting hyperplastic cells and TAMs by switching the local microenvironment from a highly permissive partner to a tumour-hostile counterpart in a murine breast cancer [16]. The anti-tumour effect of ZA also relies on the ability of the drug to impair the mesenchymal stem cells-induced TAMs recruitment to tumour sites by decreasing the expression of monocyte chemotactic protein-1 (MCP-1), thus resulting in tumour growth inhibition [30]. In addition, ZA has been reported to cooperate with different chemotherapeutic agents (e.g. docetaxel, imatinib, cisplatin) in impairing cancer cells growth *in vitro* or *in vivo* [31–34].

Although the effects of ZA are clinically well known, the molecular mechanism responsible for its effect on cancer cells are not yet well elucidated.

Here, we underscore a role of the compound on tumour microenvironment focusing on the profound impact exerted by ZA on M2 macrophages and prostate CAFs. According to our evidence, we suggest that the anti-tumour effect of ZA relies on the direct targeting of stromal and immune cells and on the prevention of their pro-tumoral skills.

Resident and recruited cells which infiltrate prostate tumours engage with cancer cells a complex network of relationships that evolve alongside malignant progression [35]. Among several cell types affecting PCa behaviour and malignancy, CAFs and macrophages exert important and overlapping roles [35, 36]. Recently, we demonstrated that CAFs and TAMs cooperate to promote PCa malignancy. Indeed, CAFs promote monocyte trans-differentiation into the M2 macrophage phenotype. In turn, this pro-tumoral subset of macrophages fosters stromal reactivity, by inducing fibroblast activation. This positive loop established between immune and stromal components is also supported by tumour cells, which participate to this cross-talk through secretion of MCP-1 and IL-6, thereby facilitating both monocyte recruitment and again differentiation into M2 macrophages [8].

Here, we demonstrate that M2 polarization is reversed by ZA treatment, which aborted IL-10 production, while it is ineffective on the recovery of the M1-polarized macrophages. In contrast, recent evidence suggest that therapeutic concentrations of ZA affect all types of macrophages, reinforcing the idea that the anti-metastatic effects of ZA are predominantly caused by modulating tumour microenvironment [37]. While M1-polarized macrophages are likely to play an anti-tumoral response, M2-polarized macrophages have been associated with a chronic inflammatory response that characterizes several highly malignant tumours [12, 38]. The ZA-dependent neutralization of M2 pro-tumoral activity brings to three main consequences: i) loss of M2 macrophages mediated enhancement of PCa cell motility; ii) reduction of stromal reactivity, due to the failure of M2 macrophages to trigger fibroblast activation; iii) inhibition of endothelial cells organization into capillary-like structures, to which cooperate both TAMs and CAFs. The overall effect is to counteract cancer cells motile spur as well as tumour vascularization, ultimately avoiding cancer cell escape from the primary site and preventing metastatic dissemination.

Besides the inhibitory effect of ZA on fibroblast activation via M2 macrophages neutralization, we also highlight a direct function of the drug in reverting the already established CAF activated phenotype. Indeed, ZA administration impairs α-SMA expression as well as its organization in stress fibers, required to enhance cell contractility. These ZA-induced effects are mainly due to the interference of the drug with the mevalonate-dependent isoprenoid metabolism [39]. Indeed, the failure in the post-translational addition of GGPP to RhoA impairs the correct localization and function of the small GTPase, thereby affecting actin cytoskeleton remodelling, stress fibers formation and ultimately cell contractility. In keeping, previous studies showed that inhibition of the mevalonate pathway by statins prevents TGF-β-induced myofibroblast transdifferentiation in human Tenon fibroblasts interfering with Rho signalling [40]. Since re-exposure to GGPP rescued ZA-dependent inhibition of a-SMA, both in terms of expression and subcellular localization, we propose RhoA geranylgeranylation as responsible for the positive effect of the mevalonate pathway on the maintenance of CAFs active state.

Alterations within the microenvironment in which the tumor develops are now recognized to be crucial during key steps of tumor progression, making tumor microenvironment components attractive candidates for therapeutic modulation. Given the tumour-supporting function elicited by CAFs and the self-reinforcing loop established between cancer and stromal cells to promote cancer cell aggressiveness, a strategy aimed at suppressing CAF activation could have interesting therapeutic implications. It has been recently reported that bisphosphonates reduce the rate of breast cancer recurrence in bone, according to a meta-analysis of randomized trials of bisphosphonates as adjuvant therapy for women with early-stage breast cancer. In particular, addition of ZA to adjuvant endocrine therapy reduced disease recurrence in bone and non-bone sites [26]. According to our evidence, a possible explanation of the efficacy of ZA could be ascribed to the effect of this bisphosphonate on CAF deactivation, through the inhibition of RhoA geranyl-geranylation. We speculate that ZA-induced stromal neutralization could interfere with the supporting role of CAFs in triggering cancer cell escaping from primary site. It is conceivable that the synergistic effect observed by combining ZA administration to conventional adjuvant endocrine therapy could rely on the double inhibitory effect on cancer cell proliferation (exerted by endocrine therapy) and metastatic dissemination (induced by ZA treatment). CAF neutralization induced by ZA may also prevent the onset of chemoresistance, thereby favoring response to conventional therapy.

Other indications strengthen the idea that interfering with CAF active state and promoting stromal normalization may be a potential antitumor approach. In particular, Mitra et al. previously reported that mimicking deregulation of miR-31, miR-214 and miR-155 resulted in the reversion of ovarian CAFs into normal fibroblasts, due to the down-regulation of several chemokines, which are known to be important for CAF function [41]. More recently, it has been proposed that prolonged exposure to hypoxia, through a prolyl hydroxylase domain protein 2-dependent stabilization of hypoxia-inducible factor-1a, counteracts a-SMA expression, ultimately leading to CAF deactivation [42].

Our results globally highlight that ZA exerts a specific targeting of stromal inflammatory cells, thus hindering their direct pro-tumoral effect and their mutual activation loop. In particular, we observe that ZA neutralizes pro-invasive and pro-angiogenic activity of M2 macrophages and fascinatingly leads to CAF deactivation, thereby strongly reducing cancer malignancy both *in vitro* and *in vivo*. These findings support ZA efficacy outside bone environment and provide an additional evidence of ZA as a potential approach to prevent cancer progression through abrogating the metastasis-promoting effects of tumour microenvironment.

## MATERIALS AND METHODS

### Materials

Unless specified, all reagents were obtained from Sigma. Antibodies against COX-2, MCSF-R and β-Actin was from Santa Cruz Biotechnology. TGF-β1, MCSF and IL-4 were from Peprotech. Rho Activator CN01 (calpeptin) was from Cytoskeleton, Inc. PVDF was from Millipore and Matrigel and GST-Rhotekin were from BD Biosciences. The ELISA kit for the quantification of IL-10 and IL-12 was from Invitrogen. ZOMETA (Zoledronic acid) was from Novartis.

### Cell cultures

Human prostate carcinoma cells (PC3 and DU145) and human umbilical vein endothelial cells (HUVEC) were obtained and authenticated by PCR/short tandem repeat (STR) analysis from the European Collection of Cell Cultures (ECACC). PC3 cells were maintained at 37°C and 5% CO2 in DMEM supplemented with 10% fetal bovine serum, while HUVEC were cultured on gelatin 1% coated dishes in EGM-2 medium (Lonza). Endothelial progenitor cells (EPCs) have been isolated from human umbilical cord blood as previously described [43, 44]. Human prostate fibroblasts (HPFs and CAFs) were isolated from surgical explantation after patient informed consent in accord with the Ethics Committee of the Azienda Ospedaliera Universitaria Careggi. Briefly, HPFs and CAFs were extracted from healthy and intratumoral regions of prostate of PCa-bearing patients with mean Gleason Score 9 (4+5) and clinical stage T3. Tissue samples were aseptically obtained from patients undergoing radical prostatectomy with final pathological examination confirmation of pT3aN0 disease. Tissues were digested overnight in 1 mg/ml collagenase I and cells were plated in DMEM containing 10% fetal bovine serum [2].

### Monocyte isolation and macrophages differentiation

Human monocytes were obtained from normal donor buffy coat by gradient centrifugation using Ficoll (Histopaque-1077). Non-adherent cells were removed and purified monocytes were incubated for 7 days in RPMI 1640 supplemented with 10% FBS and 50 ng/ml macrophage-colony stimulating factor (M-CSF) to obtain macrophages. M1 macrophages were polarized by stimulating overnight with LPS (100 ng/ml) (Peprotech) and IFNγ (100 ng/ml) (Peprotech). M2 macrophages were polarized by stimulating overnight with interleukin-4 (IL-4) (20 ng/ml) (Peprotech). M2-like macrophages were obtain by culturing monocytes for 7 days in RPMI 1640 10 % FBS with 50% of conditioned medium from PCa.

### Preparation of conditioned media

Conditioned media (CM) were obtained from untreated HPFs, CAFs, macrophages or PCa. Cells were grown to sub-confluence, then serum starved and incubated for 48h before collection of the CM. CM were harvested, clarified by centrifugation, and used freshly.

### Cell lysis and western blot analysis

Fibroblasts, PCa cells or macrophages derived from our experimental conditions were lysed for 20 min on ice in 500 μl of RIPA lysis buffer (50 mM Tris–HCl, pH 7.5, 150 mM NaCl, 1% Triton X-100, 2 mM EGTA, 1 mM sodium orthovanadate, 1 mM phenylmethanesulphonyl-fluoride, 10 mg/ml aprotinin, 10 mg/ml leupeptin). 20 μg of total proteins were loaded on SDS–PAGE, separated and transferred onto nitrocellulose. The immunoblots were incubated in 3% bovine serum albumin, 10 mmol/L Tris–HCl (pH 7.5), 1 mmol/L EDTA, and 0.1% Tween 20 for 1h at room temperature, probed first with specific antibodies and then with appropriate secondary antibodies.

### *In vitro* boyden invasion assay

PCa cell invasion was assayed with the Transwell system of Costar equipped with 8 μm-pore-size polyvinylpyrrolidone- free polycarbonate filters. Migration or invasiveness assays are distinguished by the absence (migration assay) or the presence (invasiveness assay) of a 3D barrier of Matrigel. 50 μg/cm^2^ of reconstituted Matrigel (BD Biosciences) was added to the top chamber, allowed to solidify for 1h at 37°C, and air dried for 16 h. The Matrigel barrier was rehydratated with 100 μl of Dulbecco's modified Eagle's medium for 2h at 37°C prior to use. Cells were loaded into the upper compartment (5 × 10^4^ cells in 200 μl) in serum-deprived growth medium. The Matrigel invasion chambers were placed into 24-well culture dishes containing 500 μl of the different CM from monocytes as a chemoattractant. After 24h of incubation at 37°C, non-invading cells and the Matrigel layer were mechanically removed using cotton swabs, and the microporous membrane containing the invaded cells was fixed in 96% methanol and stained with Diff-Quick staining solutions. Chemotaxis was evaluated by counting the cells that migrated to the lower surfaces of the polycarbonate filters.

### Flow cytometer analysis of apoptotic cell death

Cells undergoing apoptosis were assayed by the Annexin-V FLUOS Staining Kit (Roche Applied Sciences), according to the manufacturer's instructions. Briefly, 5×10^5^ PC3 cells were washed with ice-cold PBS and labeled for 10 min with both FITC-labelled Annexin V and Propidium iodide (PI) in 100 ml of binding buffer (HEPES-buffered saline solution with 2.5 mM CaCl_2_). Flow cytometry was performed using a FACSscan (BD Biosciences, San Jose, CA, USA). The analyzer threshold was adjusted on the flow cytometer channel to exclude most of the subcellular debris to reduce the background noise. The totality of Annexin V+/PI- (early apoptotic) and Annexin V+/PI+ cells (late apoptotic) were considered apoptotic.

### ELISA

Cytokine production in macrophages supernatants were measured by commercially available ELISA Kits (IL-10 and IL-12) according to the manufacturer's instructions (Invitrogen KHC0104/ KHC0104).

### Tube-like formation assay

All the experiments were performed using growth factor-reduced Matrigel at a concentration of 1 mg/mL. 50 μL of Matrigel were added to each well of a 96-well plate and then placed in a humidified incubator at 37°C for 30 min. HUVECs and EPCs (2×10^4^ cells/ well) were added to the Matrigel-coated plates in a final volume of 200 μL. The effects on the morphogenesis of endothelial cells were recorded after 6h with an inverted microscope equipped with CCD optics and a digital analysis system. Results were quantified by measuring the joint numbers in the field.

### Collagen gel contraction assay

1.5 × 10^5^ fibroblasts were embedded in 500 μl of DMEM solution containing 1 mg/mL collagen and cast into a well of 24-well plate (Corning, Costar). Such fibroblasts containing gel was allowed to polymerize at 37°C for 1h before detaching the gel disc from the wall using a sterile tip. Subsequently, 1 ml of serum-free DMEM with or without different concentration of ZA was added in the different wells. The floating gels were incubated at 37°C and 5% CO_2_ for 24h and the gel area were then quantified. To determine the degree of collagen gel contraction, pictures of the gels were taken and the estimated area of each gel (number of pixels) was analyzed with Adobe Photoshop^TM^.

### RhoA activity assay

HPFs or differently treated CAFs were directly lysed in RIPA buffer, the lysates were clarified by centrifugation and RhoA-GTP were quantified. Briefly, lysates were incubated with 10 μg Rhotekin-GST fusion protein (BD Biosciences) for 1h at 4°C. RhoA-GTP (on pull-down samples) and RhoA total content (on cell lysates) were then quantified by immunoblot.

### Confocal microscopy

HPFs or differentially treated CAFs were fixed in p-formaldehyde (4% v/v in PBS) for 20 min. The cells were permeabilized in Triton X-100 (0.5% v/v in PBS) for 5 min, and then incubated in horse serum (5% v/v PBS) for 30 min, and with primary antibodies against α-SMA (mouse monoclonal; 1:100; Sigma-Aldrich), overnight at 4°C. After two washing steps with PBS, cells were incubated with anti-mouse AlexaFluor 488 (1:1000; Molecular Probes) for 1h at RT in the dark. The coverslips were mounted in Gel Mount™ Aqueous Mounting Medium (Sigma-Aldrich). A Leica SP5 Confocal microscope was used for data acquisition. Images were generated with Leica SP5 Confocal Software.

### Xenograft experiments

*In vivo* experiments were performed in accordance with national guidelines and approved by the ethical committee of Animal Welfare Office of Italian Work Ministry and conform to the legal mandates and Italian guidelines for the care and maintenance of laboratory animals. 6- to 8-week-old male severe combined immunodeficient (SCID)-bg/bg mice (Charles River Laboratories International) were injected s.c. with 1×10^6^ PC3 cells plus 0.5×10^6^ CAFs, both in the right and left lateral flanks. Mice received repeated courses of intra-peritoneal saline solution (control mice) or 100 μg/kg of ZA, once a week for 6 weeks. Animals (5 per group) were monitored daily. Tumour size was measured every 2 to 3 days by a caliper; tumor volumes were determined by the length (L) and the width (W): V = (LW2)/2. Mice were sacrificed after 6 weeks. Lungs were inspected for metastatic nodules by histological analyses.

### Histology

Lungs were fixed in 4% (vol/vol) phosphate-buffered formalin and paraffin embedded. Five longitudinal sections of each lung per mice (5 μm thick) were mounted on positively charged slides. Tissue sections were deparaffinized, re-hydrated and stained with hematoxylin-eosin staining. Metastases were counted using NIS ELEMENTS_F-2.20 software combined with a microscope Nikon Eclipse 50i.

### Statistical analysis

Data are presented as mean ± SD from at least three independent experiments. Statistical analysis of the data was performed using GraphPad Prism and the relevant analyses are indicated in the Figure Legends.

## SUPPLEMENTARY FIGURES


