# Supplementary material for: Zoledronic acid impairs stromal reactivity by inhibiting M2-macrophages polarization and prostate cancer-associated fibroblasts
Source: Oncotarget. 2016 May 20;8(1):118–32. doi: 10.18632/oncotarget.9497 (PMC5352046; doi:10.18632/oncotarget.9497)
Supplement: Supplementary file 1 [file oncotarget-08-118-s001.pdf]

Zoledronic acid impairs stromal reactivity by inhibiting M2-macrophages polarization and prostate cancer-associated fibroblasts

SUPPLEMENTARY FIGURES

A

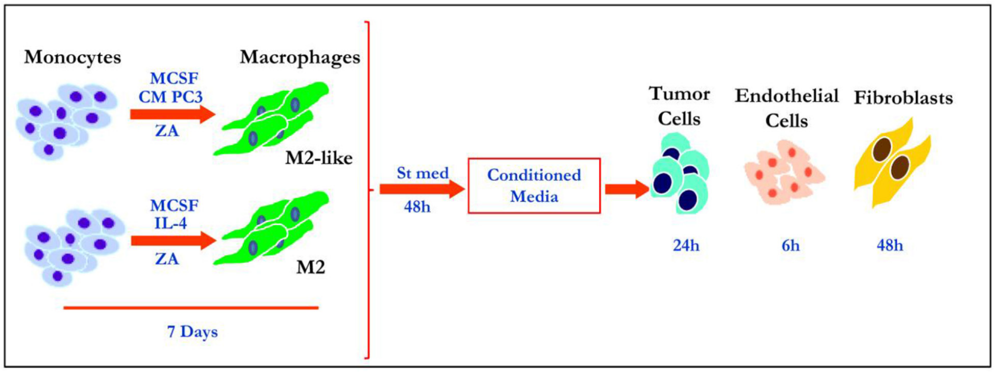

B

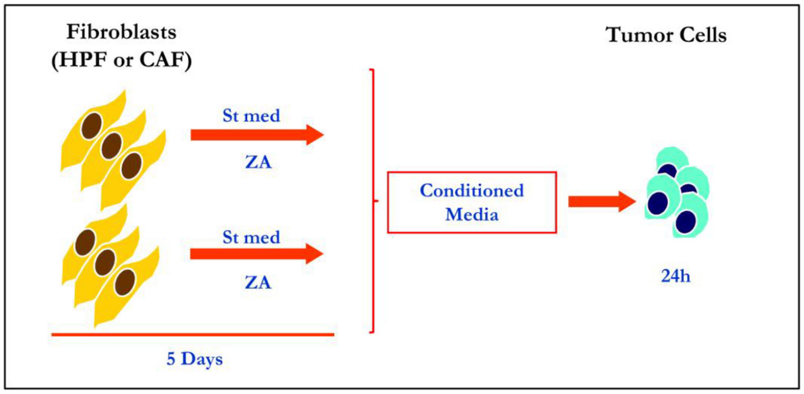

Supplementary Figure S1: Experimental design A. Human monocytes isolated from normal donor buffy coat were cultured for 7 days with M-CSF (50ng/ml) and then polarized into M2 macrophages by stimulating with IL-4 for 24h. Alternatively, monocytes were stimulated with CM from PC3 for 7 days to obtain M2-like macrophages. ZA was administrated during differentiation at different concentrations and then macrophages were serum-starved for 48h to obtain the corresponding CM. **B.** Subconfluent HPFs and CAFs were serum starved and treated for 5 days with different concentration of ZA to obtain the corresponding CM.

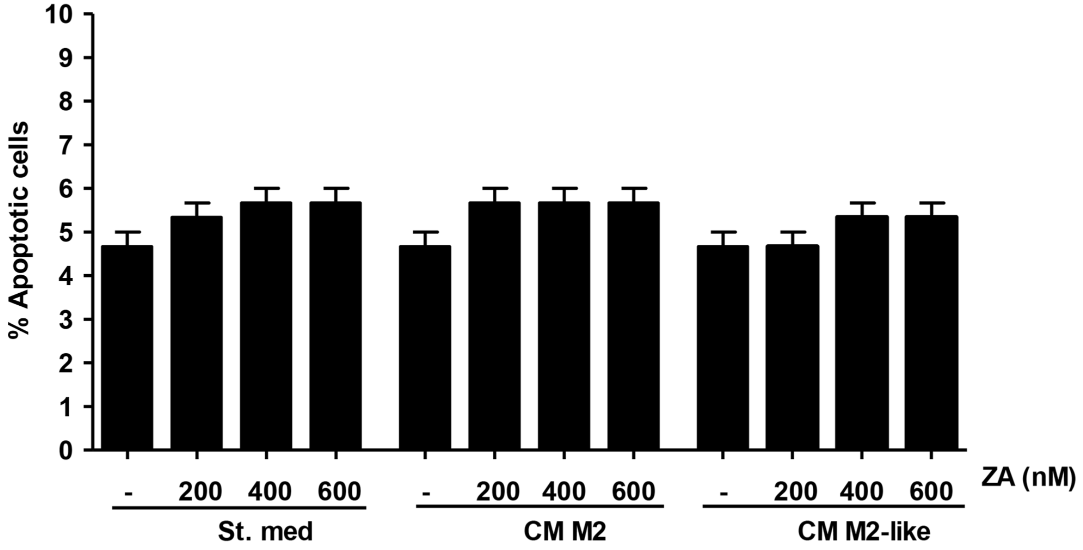

Supplementary Figure S2: Monocytes were differentiated for 7 days with M-CSF and then polarized into M2 macrophages by stimulating with IL-4 for 24h. Alternatively, monocytes were stimulated with CM from PC3 for 7 days to obtain M2-like macrophages. ZA was administrated during differentiation at different concentrations and then macrophages were serum-starved for 48h to obtain the corresponding CM. PC3 cells were incubated for 24h with CM from the above differentiated macrophages (treated or not with ZA), or serum starved as a control (treated or not with ZA). The percentage of apoptotic cells was evaluated using the Annexin V-Pi staning Kit.

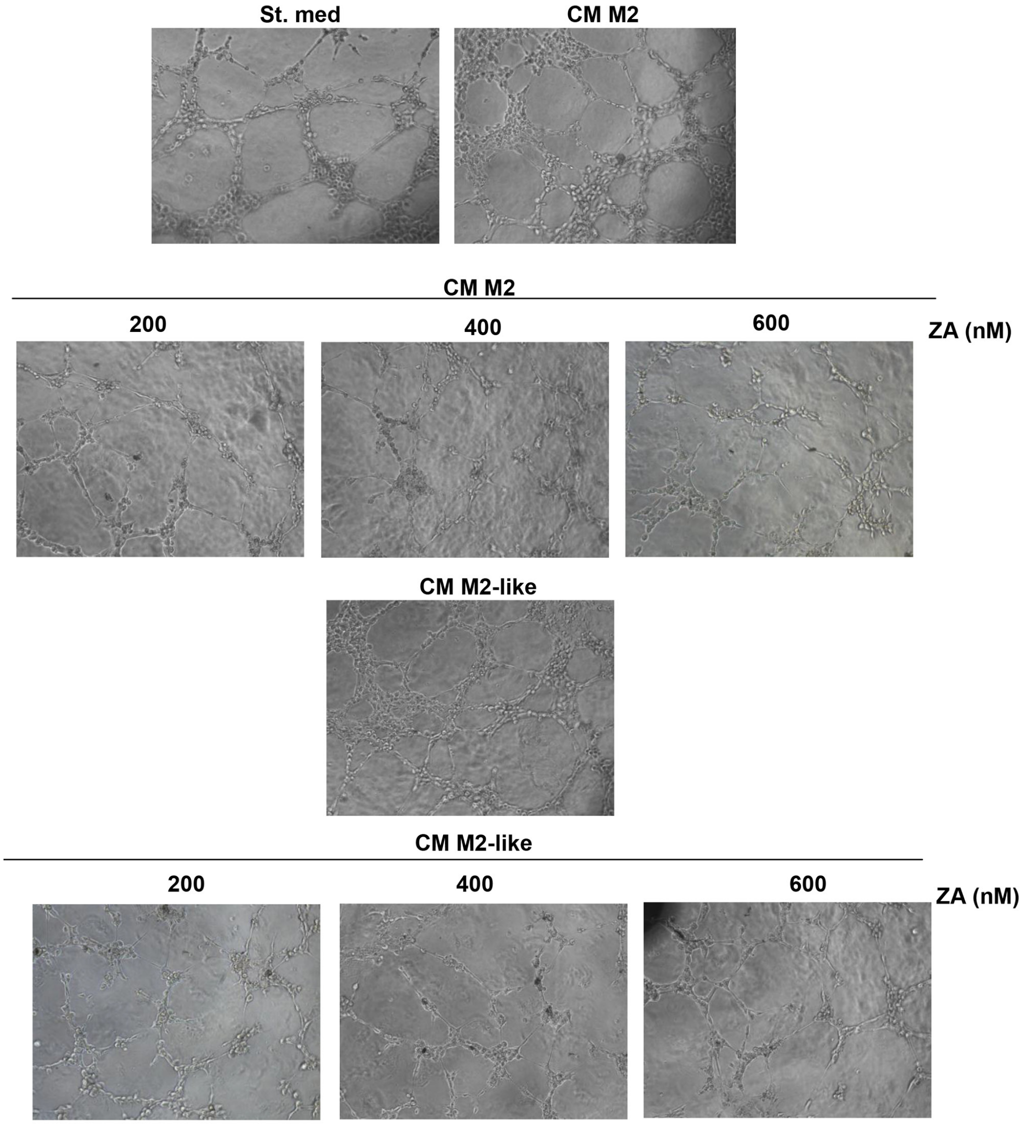

Supplementary Figure S3: M2 and M2-like macrophages were treated with different concentrations of ZA during differentiation and then were serum-starved for 48h to obtain the corresponding CM. HUVEC were treated with CM from the above differentiated macrophages (treated or not with ZA) and *in vitro* angiogenesis was evaluated by capillary morphogenesis assay. Representative photographs of tube formation are shown.

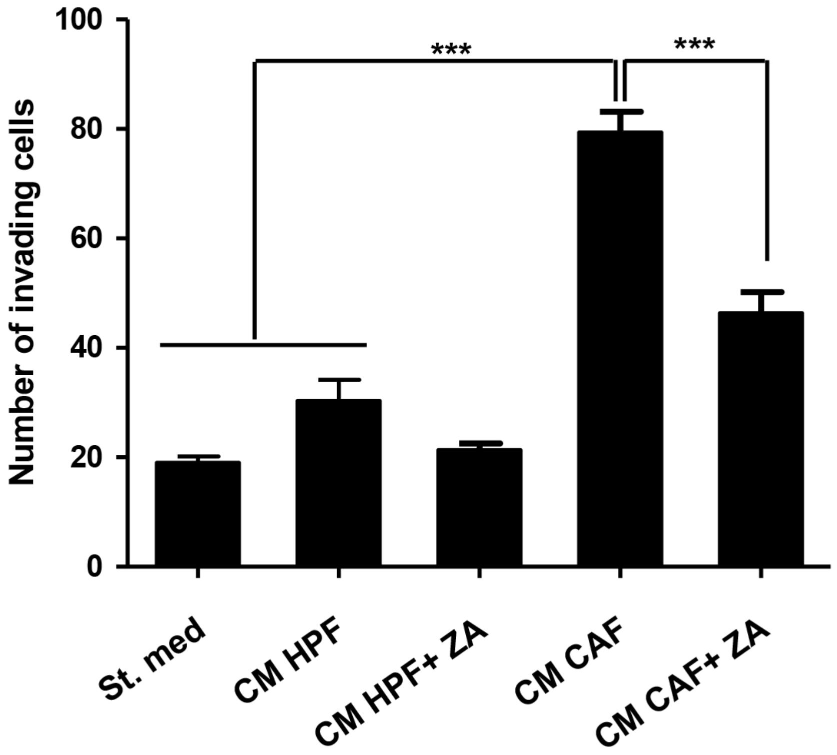

Supplementary Figure S4: DU145 cells were incubated for 24 h with CM from HPFs and CAFs treated with 200 nM of ZA and then allowed to invade for additional 24 h toward medium containing 10% serum as chemoattractant. Invading cells were counted, and a bar graph, representative of six randomly chosen fields, is shown. 1-way ANOVA, Dunnett's corrected, ***p< 0,001 *vs* CM CAF.

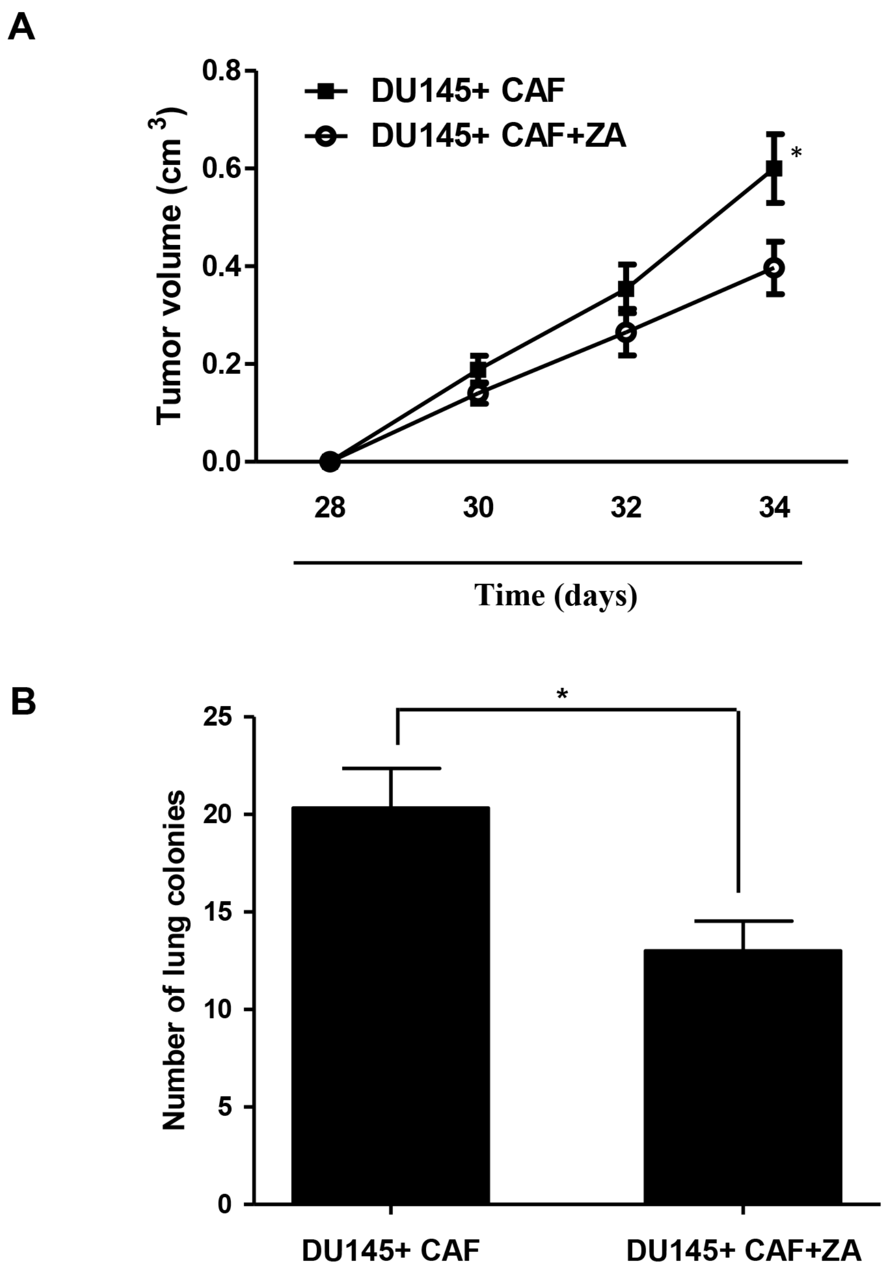

Supplementary Figure S5: A. Primary tumor growth in SCID bg/bg mice of DU145 cells subcutaneously injected in both the lateral flanks with CAFs, mice were treated once per week for 6 weeks with PBS (control) or 100 μg/kg of ZA. The onset and volume of the primary tumor are reported in the growth curve. 2-way ANOVA, Bonferroni's corrected *p<0,05 *vs* untreated. **B.** Animals were monitored at 2-day intervals and were sacrificed after 6 weeks. Lungs were inspected with the aid of a microscope and micrometastases were counted. Student t-test, *p<0,0436 *vs* DU145+CAF.
